# Glucose and Prolactin Monitoring in Children and Adolescents Initiating Antipsychotic Therapy

**DOI:** 10.1089/cap.2018.0013

**Published:** 2018-09-14

**Authors:** Yasuyuki Okumura, Masahide Usami, Takashi Okada, Takuya Saito, Hideki Negoro, Noa Tsujii, Junichi Fujita, Junzo Iida

**Affiliations:** ^1^ Research Department, Institute for Health Economics and Policy, Association for Health Economics Research and Social Insurance and Welfare, Tokyo, Japan.; ^2^ Department of Psychiatry and Behavioral Science, Tokyo Metropolitan Institute of Medical Science, Tokyo, Japan.; ^3^ Department of Child and Adolescent Psychiatry, Kohnodai Hospital, National Center for Global Health and Medicine, Ichikawa, Japan.; ^4^ Department of Child and Adolescent Psychiatry, Nagoya University Graduate School of Medicine, Nagoya, Japan.; ^5^ Department of Child and Adolescent Psychiatry, Faculty of Medicine, Hokkaido University, Sapporo, Japan.; ^6^ Department of Professional Development in Education, Graduate School of Professional Development in Education, Nara University of Education, Nara, Japan.; ^7^ Department of Neuropsychiatry, Kindai University Faculty of Medicine, Osaka, Japan.; ^8^ Department of Child Psychiatry, Yokohama City University Hospital, Yokohama, Japan.; ^9^ Department of Human Development, Faculty of Nursing, Nara Medical University, Kashihara, Japan.

**Keywords:** antipsychotics, quality of care, diabetes, side effects, metabolic monitoring

## Abstract

***Objective:*** We aimed to evaluate glucose and prolactin monitoring in children and adolescents initiating antipsychotic therapy using a nationwide claims database.

***Methods:*** A retrospective 15-month cohort study was conducted using the National Database of Health Insurance Claim Information and Specified Medical Checkups in Japan. Patients aged ≤18 years, who were newly prescribed antipsychotics between April 2014 and March 2015, were followed up for 450 days. Outcomes were the use of glucose and prolactin testing through 15 months after drug initiation (index date) with consideration of persistence with antipsychotic therapy. The incidence proportion of patients monitored was assessed within the following four time windows: baseline (between 30 days before the index date and the index date), at 1–3 months (between 1 and 90 days after the index date), at 4–9 months (between 91 and 270 days after the index date), and at 10–15 months (between 271 and 450 days after the index date).

***Results:*** Of 43,608 new users in 6620 medical institutions, the percentage of persistent antipsychotic users was 46.4% at 90 days, 29.7% at 270 days, and 23.8% at 450 days after the index date. The proportion of patients who received monitoring within the baseline period was 13.5% (95% confidence interval [CI], 13.2–13.8) for glucose and 0.6% (95% CI, 0.5–0.6) for prolactin, respectively. The proportion of patients who received glucose monitoring at all time windows decreased to 0.9%. The proportion of patients who received prolactin monitoring by the second time window decreased to 0.1%.

***Conclusions:*** Our study shows that monitoring for glucose and prolactin is infrequent in children and adolescents initiating antipsychotic therapy. Strategies for physicians, patients, and guardians are needed to overcome the barriers in glucose and prolactin monitoring.

## Introduction

Antipsychotics have been increasingly prescribed to children and adolescents worldwide (Hsia and Maclennan 2009; Okumura et al. 2014; Olfson et al. 2014). The initiation of antipsychotics is associated with an increased risk of type II diabetes in children and adolescents (Bobo et al. 2013; Rubin et al. 2015; Galling et al. 2016). The risk of type II diabetes increases with a cumulative dose of antipsychotics (Bobo et al. 2013). Therefore, antipsychotic prescribers are recommended to routinely monitor children and adolescents for metabolic abnormalities (American Academy of Child and Adolescent Psychiatry 2011; Pringsheim et al. 2011; Galling et al. 2016; Pisano et al. 2016). This recommendation is in concordance with the monitoring protocol from the American Diabetes Association and collaborative associations (American Diabetes Association et al. 2004), which recommends that fasting plasma glucose should be assessed at baseline, 3 months, and 12 months after drug initiation, primarily among adult users of second-generation antipsychotics.

Despite the caution about diabetes risk for antipsychotics, to the best of our knowledge, there have been only four studies of metabolic monitoring patterns among children and adolescents initiating antipsychotic therapy. So far, no study has been conducted outside the United States. In a cohort study of 5370 Medicaid beneficiaries aged 6–17 years, who initiated second-generation antipsychotic treatment between 2004 and 2006, 32% received glucose screening between 30 days before and 180 days after drug initiation (Morrato et al. 2010). In a cohort study of 16,304 patients aged 2–18 years, who initiated second-generation antipsychotic treatment between 2006 and 2011 in the Mini-Sentinel Distributed Database, 12% underwent a glucose test between 90 days before and 3 days after initiation (Raebel et al. 2014). In a cohort study of 52,407 commercially insured beneficiaries aged 5–18 years, who initiated second-generation antipsychotic treatment between 2003 and 2011, the proportion of patients who received glucose monitoring was 16% in the 180 days before drug initiation and 16% in the 180 days after drug initiation (Connolly et al. 2015). In a cohort study of 1023 commercially insured beneficiaries aged 0–17 years, who initiated second-generation antipsychotic treatment between 2002 and 2011, the proportion of patients receiving glucose monitoring was 8% in the baseline period between 84 days before and 14 days after drug initiation (Delate et al. 2014). In the same study, the proportion was 12% in the follow-up period from the date of baseline monitoring (or 15 days after drug initiation when baseline monitoring was not performed) to 84 days after drug initiation (Delate et al. 2014).

However, there are several methodological limitations to these studies in which the number of time windows was set to one or two (e.g., 30 days before to 180 days after the drug initiation), and the status of metabolic monitoring for all patients initiating antipsychotics was assessed within the time windows. The small number of time windows (i.e., two time windows at most) may have restricted the ability to assess the status of “regular” metabolic monitoring during antipsychotic therapy. In addition, some previous studies included patients who initiated antipsychotic therapy but, who did not require glucose monitoring because of treatment discontinuation.

Furthermore, the status of prolactin monitoring has not been assessed in previous studies. The initiation of risperidone, olanzapine, and the majority of first-generation antipsychotics is associated with an increased risk of hyperprolactinemia, although aripiprazole and quetiapine appear to present a better hyperprolactinemia profile (De Hert et al. 2011; Montejo et al. 2016; Pisano et al. 2016). Hyperprolactinemia may lead to amenorrhea, galactorrhea, and gynecomastia in the short term after drug initiation. Potential long-term adverse consequences of hyperprolactinemia include bone mineral density loss, fractures, and breast cancer, although there is no definitive evidence on the long-term risk of hyperprolactinemia (Bushe et al. 2010).

There is some controversy in the current guidelines on recommendations for regular monitoring of serum prolactin because of lack of evidence on the long-term consequences of chronic elevation of prolactin in the absence of prolactin-related symptoms. The U.S. guideline has issued a recommendation against regular prolactin monitoring for antipsychotic users in the absence of prolactin-related symptoms (American Academy of Child and Adolescent Psychiatry 2011), while the Canadian guideline strongly recommends regular prolactin monitoring (Pringsheim et al. 2011).

Although there is no guideline for the use of antipsychotics specific to children and adolescents in Japan, we believe that the benefits of regular prolactin monitoring may outweigh the risks. This is because children and adolescents are more sensitive to the consequence of hyperprolactinemia, which indicates that antipsychotics should be used judiciously due to the risk of bone mineral density loss (Montejo et al. 2016). Thus, the uncertainty of evidence on the long-term risk of hyperprolactinemia is not a reason for dismissing the recommendation for regular monitoring of serum prolactin. In addition, children and adolescents often cannot express their symptoms adequately and they sometimes have asymptomatic hyperprolactinemia (Pringsheim et al. 2011). Moreover, the baseline risk of hyperprolactinemia might be high in Japan due to the fact that prolactin-raising antipsychotics (i.e., risperidone and first-generation antipsychotics) are commonly prescribed to children and adolescents (Inoue et al. 2017). Furthermore, serum prolactin can be measured at the same time glucose monitoring is done, without an additional blood draw, although this leads to a slight increase in the blood sample volume and an additional cost of 980 yen (∼10 U.S. dollars).

To overcome these limitations, we conducted a 15-month cohort study and included a consideration of persistence with antipsychotic therapy. The primary objective of this study was to evaluate glucose and prolactin monitoring for 15 months in children and adolescents initiating antipsychotic therapy using a nationwide claims database in Japan.

## Methods

### Data source

A retrospective cohort study was conducted using the National Database of Health Insurance Claim Information and Specified Medical Checkups (NDB) in Japan, which has a universal healthcare system. The Ministry of Health, Labour and Welfare (MHLW) in Japan has collected almost all claims since April 2009 (Ministry of Health, Labour and Welfare 2013), with the exception of those who paid all medical fees without using public health insurance. The NDB includes information on institution, patient, and procedural characteristics such as institution codes, patient identification numbers, sex, age group, date of procedures, procedural codes, date of prescriptions, drug codes, days of drug supply, and dosage. The NDB includes two types of patient identification numbers: the ID1 is generated from the insurance identification number, birth date, and sex and the ID2 is generated from name, birth date, and sex. Both identification numbers have limitations in the traceability, although these limitations are less likely to affect the population of children and adolescents. For example, the ID1 cannot follow up on patients who change their jobs and ID2 cannot follow up on patients who change their family names. The NDB has been used in several studies (Maeda et al. 2018; Okumura and Nishi 2017; Okumura et al. 2017). Our study protocol was reviewed and approved by the institutional review board at the Institute of Health Economics and Policy.

### Patient selection

We identified all patients aged ≤18 years, who were prescribed antipsychotics between April 2014 and March 2015. We included an exhaustive list of antipsychotics with the exception of clozapine and a chlorpromazine-promethazine-phenobarbital combination drug (Supplementary Table S1; Supplementary Data are available online at www.liebertpub.com/cap). To increase traceability, we implemented a new algorithm that used both the ID1 and ID2, referred to as “ID0,” for patient identification (Kubo et al. 2018). To focus on new users of antipsychotics, we identified the index date, which is the date of first prescription (between April 2014 and March 2015), and included those who enrolled in the NDB at least 180 days before the index date, while excluding those who obtained a prescription within 180 days before the index date. We also excluded patients with a preexisting definitive diagnosis of diabetes according to the diagnostic codes in the Charlson Index (ICD-10 codes: E10.1–E10.5, E10.9, E11.1–E11.5, E11.9, E13.1–E13.5, E13.9, E14.1–E14.5, and E14.9) (Sundararajan et al. 2007). We excluded patients who had incomplete claim information during 180 days before and 480 days after the index date, in which the status of prescription and screening was not recorded. To ensure a follow-up period of at least 450 days (with a 30-day grace period) after the index date, we included patients who enrolled in the NDB at least 480 days after the index date.

### Outcomes

Outcomes of interest for this study were the use of glucose and prolactin testing through 15 months after drug initiation. In this study, glucose testing refers to either blood glucose or hemoglobin A1c (HbA1c) tests. The procedural codes used for blood glucose, HbA1c, and prolactin testing were 160019410, 160010010, and 160032310, respectively. In this study, four time windows were defined as follows: baseline (between 30 days before the index date and the index date), 1–3 months (between 1 and 90 days after the index date), 4–9 months (between 91 and 270 days after the index date), and 10–15 months (between 271 and 450 days after the index date) (Supplementary Fig. S1).

### Covariates

We extracted information on provider, demographic, and medication characteristics at the index date. These covariates were selected on the basis of evidence from previous studies and clinical experiences (Morrato et al. 2010; Raebel et al. 2014; Connolly et al. 2015). Provider characteristics included medical institution code, provider type (clinic/hospital), setting (inpatient/ambulatory care), and prescriber type (psychiatrist/nonpsychiatrist). In Japan, hospitals are defined as medical institutions with ≥20 beds. Demographic characteristics included sex (boys/girls) and age (0–3/4–6/7–12/13–15/16–18 years). Medication characteristics included individual antipsychotics (aripiprazole/chlorpromazine/haloperidol/olanzapine/prochlorperazine/quetiapine/risperidone/sulpiride/others), types of antipsychotics (first generation/second generation/both), and the chlorpromazine-equivalent dosage of antipsychotics (<100/100–299/300–499/≥500 mg) (Inada and Inagaki 2015) (Supplementary Table S1). The categories of the individual antipsychotics were based on the top eight most frequently prescribed antipsychotics. Patients treated with multiple antipsychotics were classified into an “other” category. The categories of the dosage of the antipsychotics were based on a previous study (Deb et al. 2015).

### Statistical analyses

First, descriptive analyses of overall and persistent antipsychotic users were conducted. During the follow-up period of 450 days after drug initiation, discontinuity of antipsychotic therapy was designated when an antipsychotic prescription was not refilled within an interval defined by the days of drug supply plus a grace period of 30 days (Dezii 2001). If there was no prescription for an antipsychotic within the interval, patients were assigned a discontinuation date according to the last day of the drug supply. For example, if a patient received a prescription of antipsychotics for 14 days and did not receive an additional prescription within the subsequent 30 days, then the date of treatment discontinuation for the patient was set at 14 days after drug initiation. Patients were grouped according to persistence with antipsychotic therapy: 3-month (at least 90 days after the index date), 9-month (at least 270 days after the index date), and 15-month (at least 450 days after the index date) persistent users (Supplementary Fig. S1).

Second, the incidence proportion for those who received glucose and prolactin testing and their 95% confidence intervals (CIs) were estimated by time windows. For example, the incidence proportion of those monitored at the 1- to 3-month period (1 and 90 days after the index date) was calculated based on the number of patients who received monitoring between 1 and 90 days after the index date among the 3-month (at least 90 days after the index date) persistent users.

The incidence proportion of those who received regular monitoring of glucose and prolactin testing was also estimated using a time window. Regular (consecutive) monitoring was defined as monitoring that was consecutively performed at each time window. For example, patients who received regular monitoring at the 4- to 9-month period (between 91 and 270 days after the index date) were 9-month (at least 270 days after the index date) persistent users who received glucose test at baseline, at 1–3 months, and at 4–9 months. Because the baseline duration (31 days) and 1–3 months (90 days) are much shorter than 4–9 months (180 days) and 10–15 months (180 days), we created an additional time window of 0–3 months that combined the baseline and 1- to 3-month periods (between 30 days before and 90 days after the index date). Following this, we calculated the incidence proportion of regular monitoring at all the three time windows.

Third, to assess the time to initial monitoring for glucose and prolactin between 30 days before and 450 days after the index date, we used the Aalen-Johansen estimator, which can account for the competing risk (Beyersmann et al. 2011). Nonpersistence of antipsychotic therapy during the follow-up period was considered a competing risk event. Patients who did not receive monitoring were censored at 450 days after the index date.

Fourth, to compare the incidence proportions of those who received baseline monitoring, we used generalized estimating equations with a Poisson distribution and a log-link function to account for correlated data structure (patients clustered within medical institutions) (Hanley et al. 2003). All covariates were simultaneously entered into the models, with the exception of individual antipsychotics and types of antipsychotics. To avoid multicollinearity, individual antipsychotics were used only in the main analyses, while types of antipsychotics were used only in the sensitivity analyses. Crude and adjusted incidence proportion ratios (IPRs) and their 95% CI were derived from models. Adjusted incidence proportions were estimated by averaging the predicted values from the models across the covariate distribution (Lenth 2016).

All statistical analyses were performed using R version 3.4.1 (R Foundation for Statistical Computing) with the geepack package (Højsgaard 2016). Significance level was set at 5%. Cells with a count ≤9 are not reported according to the cell size suppression policy of the database (Ministry of Health, Labour and Welfare 2013).

## Results

### Study population

During the study period, 43,608 children and adolescents with newly initiated antipsychotic therapy in 6620 medical institutions were identified and included (Supplementary Fig. S2). The annual number of new antipsychotic users in an institution ranged 1–445 with a median of 2 (interquartile range: 1–5). Table 1 shows characteristics of the study participants. Of these, 57.8% received treatments in clinics, 94.1% received ambulatory care, and 55.1% visited psychiatrists. The boy:girl ratio was 1:0.7 and the largest group was 16–18 years (35.9%) of age. The top three most common antipsychotics were risperidone (30.3%), aripiprazole (23.6%), and sulpiride (21.4%). The percentage of persistent antipsychotic users was 46.4% at 90 days, 29.7% at 270 days, and 23.8% at 450 days after the index date (Supplementary Fig. S3 and Table 1).

**Table 1.** Sample Characteristics

			*Persistent user*^a^					
	*Total (*N* = 43,608)*		*3 Month*^b^*(*N* = 20,370)*		*9 Month*^c^*(*N* = 12,964)*		*15 Month*^d^*(*N* = 10,378)*	
*Characteristics*	n	*%*	n	*%*	n	*%*	n	*%*
Provider type								
Clinic	25,222	57.8	11,058	54.3	6774	52.3	5360	51.6
Hospital	18,386	42.2	9312	45.7	6190	47.7	5018	48.4
Setting								
Inpatient	2567	5.9	295	1.4	180	1.4	149	1.4
Ambulatory	41,041	94.1	20,075	98.6	12,784	98.6	10,229	98.6
Prescriber								
Nonpsychiatrist	19,601	44.9	7428	36.5	4906	37.8	4002	38.6
Psychiatrist	24,007	55.1	12,942	63.5	8058	62.2	6376	61.4
Sex								
Boys	25,192	57.8	13,004	63.8	8703	67.1	7082	68.2
Girls	18,416	42.2	7366	36.2	4261	32.9	3296	31.8
Age, years								
0–3	445	1.0	90	0.4	71	0.5	61	0.6
4–6	2927	6.7	1428	7.0	1104	8.5	972	9.4
7–12	13,217	30.3	7573	37.2	5314	41.0	4408	42.5
13–15	11,378	26.1	5106	25.1	3027	23.3	2332	22.5
16–18	15,641	35.9	6173	30.3	3448	26.6	2605	25.1
Individual antipsychotics								
Aripiprazole	10,300	23.6	6342	31.1	4131	31.9	3250	31.3
Chlorpromazine	2087	4.8	139	0.7	75	0.6	53	0.5
Haloperidol	1173	2.7	447	2.2	280	2.2	227	2.2
Olanzapine	1446	3.3	741	3.6	465	3.6	372	3.6
Prochlorperazine	1743	4.0	33	0.2	≤9	≤0.1	≤9	≤0.1
Quetiapine	1075	2.5	498	2.4	289	2.2	230	2.2
Risperidone	13,205	30.3	7899	38.8	5542	42.7	4628	44.6
Sulpiride	9317	21.4	2736	13.4	1195	9.2	818	7.9
Others	3262	7.5	1535	7.5	979	7.6	794	7.7
Types of antipsychotics								
FGA	16,015	36.7	3954	19.4	1937	14.9	1398	13.5
SGA	27,206	62.4	16,196	79.5	10,884	84.0	8858	85.4
Both	387	0.9	220	1.1	143	1.1	122	1.2
Chlorpromazine-equivalent dosage, mg								
0–99	35,328	81.0	16,475	80.9	10,306	79.5	8170	78.7
100–299	7265	16.7	3535	17.4	2393	18.5	1974	19.0
300–499	498	1.1	246	1.2	185	1.4	159	1.5
≥500	517	1.2	114	0.6	80	0.6	75	0.7

^a^ Cells with counts ≤9 cannot be reported according to the cell size suppression policy of the database. Percentages for the cell with counts ≤9 are displayed with the numerator equal to 9 along with the ≤ signs.

^b^ 3-Month persistent users were defined as those who continued antipsychotic therapy at least 90 days after the index date.

^c^ 9-Month persistent users were defined as those who continued antipsychotic therapy at least 270 days after the index date.

^d^ 15-Month persistent users were defined as those who continued antipsychotic therapy at least 450 days after the index date.

FGA, first-generation antipsychotics; SGA, second-generation antipsychotics.

### Incidence proportion of monitoring

Table 2 shows the incidence proportion of those who received monitoring for glucose and prolactin testing. The incidence proportion of monitoring within baseline period was 13.5% (95% CI, 13.2–13.8) for glucose and 0.6% (95% CI, 0.5–0.6) for prolactin, respectively. The incidence proportion of those who received regular monitoring for glucose decreased to 2.3% within two time windows (baseline and 1- to 3-month periods); to 1.3% within three time windows (baseline, 1- to 3-month, and 4- to 9-month periods); and to 0.9% within four time windows (baseline, 1- to 3-month, 4- to 9-month, and 10- to 15-month periods). The incidence proportion of those who received regular monitoring for prolactin decreased to only 0.1% within two time windows (baseline and 1- to 3-month periods). When using three time windows (0- to 3-month, 4- to 9-month, and 10- to 15-month periods), the incidence proportion of those who received regular monitoring was 3.8% for glucose and 0.2% for prolactin.

**Table 2.** Monitoring for Glucose and Prolactin Testing by Time Window

		*Glucose*		*Prolactin*	
*Time window (days from index date)*	*No. of persistent user*	*Percentage of monitoring (95% CI)*^a^	*Percentage of regular monitoring (95% CI)*^b,c^	*Percentage of monitoring (95% CI)*^a^	*Percentage of regular monitoring (95% CI)*^b,c^
Baseline (−30 to 0 day)	43,608	13.5 (13.2–13.8)	—	0.6 (0.5–0.6)	—
1–3 Months (1–90 days)	20,370	10.8 (10.4–11.2)	2.3 (2.1–2.6)	1.1 (1.0–1.3)	0.1 (0.1–0.2)
4–9 Months (91–270 days)	12,964	15.7 (15.1–16.3)	1.3 (1.1–1.5)	2.1 (1.8–2.3)	≤0.1
10–15 Months (271–450 days)	10,378	15.6 (14.9–16.3)	0.9 (0.7–1.1)	1.9 (1.6–2.2)	≤0.1

^a^ Percentage of monitoring within the time window.

^b^ Percentage of regular (consecutive) monitoring until the time window.

^c^ Cells with counts ≤9 cannot be reported according to the cell size suppression policy of the database. The incidence proportion of those with cell counts ≤9 is displayed with the numerator equal to 9 along with the ≤ signs.

CI, confidence interval.

### Time to initial monitoring

**FIG. 1.**
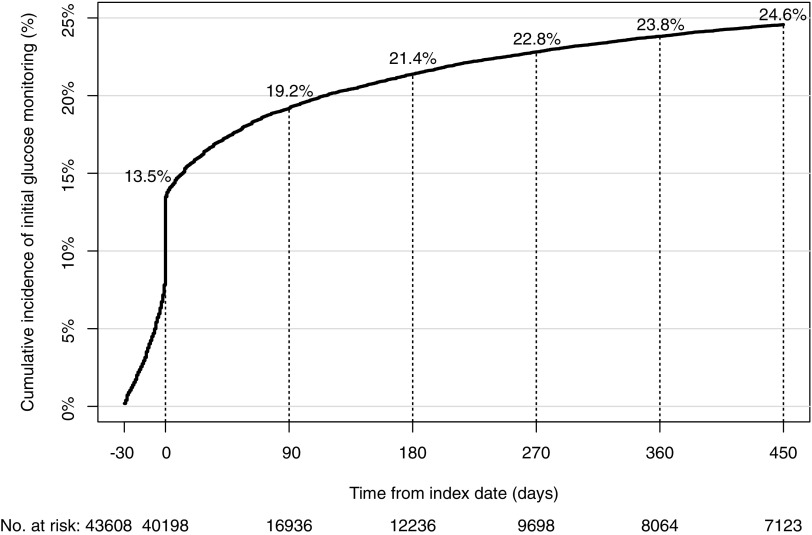
Cumulative incidence of initial glucose monitoring.

**FIG. 2.**
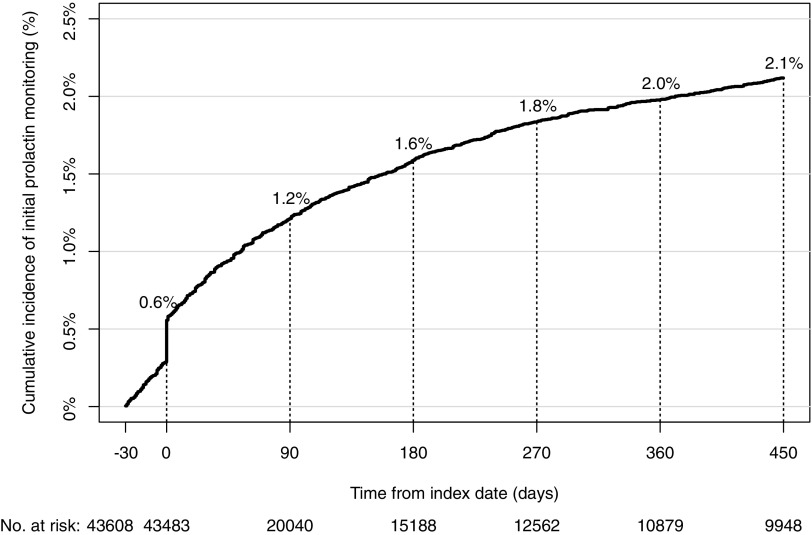
Cumulative incidence of initial prolactin monitoring.

The percentage of initial glucose monitoring increased steeply to 13.5% at the index date and increased gradually from 21.4% at 180 days to 24.6% at 450 days after the index date (Fig. 1). The percentage of initial prolactin monitoring also increased steeply to 0.6% at the index date and increased gradually from 1.6% at 180 days to 2.1% at 450 days after the index date (Fig. 2).

### Correlates of baseline monitoring

Table 3 presents results from the generalized estimating equations. There were several determinants of baseline monitoring. For example, patients with a prescription for olanzapine were more likely to receive a glucose test than those with a prescription for aripiprazole (adjusted IP, 25.4% vs. 17.3%; adjusted IPR, 1.47). Girls were more likely to receive a prolactin test than boys (adjusted IP, 0.9% vs. 0.3%; adjusted IPR, 2.73). However, the incidence proportion of those who received monitoring within the baseline period was <30% for glucose and <1% for prolactin in almost all subgroups. Sensitivity analyses showed similar results (Supplementary Table S2).

**Table 3.** Incidence and Correlates of Metabolic and Prolactin Monitoring at Baseline

	*Glucose*^a^				*Prolactin*^a^			
	*Crude*		*Adjusted*^b^		*Crude*		*Adjusted*^b^	
*Characteristics*	*IP*	*IPR (95% CI)*	*IP*	*IPR (95% CI)*	*IP*	*IPR (95% CI)*	*IP*	*IPR (95% CI)*
Provider type								
Clinic	0.102	Ref.	0.217	Ref.	0.004	Ref.	0.004	Ref.
Hospital	0.180	1.77 (1.39–2.25)	0.267	1.23 (0.96–1.57)	0.007	1.66 (0.95–2.90)	0.008	2.00 (1.01–3.94)
Setting								
Inpatient	0.738	Ref.	0.513	Ref.	0.012	Ref.	0.011	Ref.
Ambulatory	0.097	0.13 (0.11–0.15)	0.113	0.22 (0.13–0.37)	0.005	0.43 (0.27–0.67)	0.003	0.27 (0.14–0.53)
Prescriber								
Nonpsychiatrist	0.182	Ref.	0.249	Ref.	0.005	Ref.	0.005	Ref.
Psychiatrist	0.097	0.53 (0.44–0.64)	0.232	0.93 (0.79–1.10)	0.006	1.39 (0.94–2.05)	0.007	1.41 (0.92–2.16)
Sex								
Boys	0.112	Ref.	0.222	Ref.	0.003	Ref.	0.003	Ref.
Girls	0.166	1.49 (1.39–1.59)	0.260	1.17 (1.12–1.23)	0.009	3.19 (1.81–5.60)	0.009	2.73 (1.72–4.33)
Age, years								
0–3	0.494	2.90 (2.30–3.65)	0.197	0.61 (0.50–0.75)	≤0.020	—	—	—
4–6	0.160	0.94 (0.58–1.50)	0.229	0.71 (0.57–0.89)	≤0.003	—	—	—
7–12	0.076	0.45 (0.34–0.58)	0.193	0.60 (0.51–0.70)	0.004	0.47 (0.22–1.00)	0.006	0.83 (0.37–1.83)
13–15	0.134	0.78 (0.73–0.84)	0.284	0.88 (0.83–0.94)	0.005	0.64 (0.45–0.90)	0.005	0.73 (0.51–1.04)
16–18	0.171	Ref.	0.323	Ref.	0.008	Ref.	0.007	Ref.
Individual antipsychotics								
Aripiprazole	0.074	Ref.	0.173	Ref.	0.008	Ref.	0.012	Ref.
Chlorpromazine	0.491	6.61 (4.83–9.06)	0.477	2.76 (1.23–6.19)	≤0.004	—	—	—
Haloperidol	0.260	3.51 (2.73–4.50)	0.212	1.22 (0.92–1.61)	≤0.008	—	—	—
Olanzapine	0.158	2.14 (1.69–2.69)	0.254	1.47 (1.16–1.86)	≤0.006	—	—	—
Prochlorperazine	0.362	4.88 (3.54–6.72)	0.270	1.56 (1.05–2.33)	≤0.005	—	—	—
Quetiapine	0.136	1.83 (1.43–2.35)	0.227	1.31 (0.99–1.74)	≤0.008	—	—	—
Risperidone	0.061	0.83 (0.69–1.00)	0.140	0.81 (0.69–0.96)	0.002	0.33 (0.16–0.70)	0.004	0.36 (0.15–0.83)
Sulpiride	0.170	2.29 (1.88–2.80)	0.352	2.03 (1.65–2.50)	0.008	1.06 (0.53–2.12)	0.013	1.12 (0.60–2.07)
Others	0.118	1.60 (1.24–2.06)	0.199	1.15 (0.91–1.45)	0.007	0.97 (0.40–2.37)	0.009	0.74 (0.32–1.71)
Chlorpromazine-equivalent dosage, mg								
0–99	0.122	Ref.	0.203	Ref.	0.006	Ref.	0.006	Ref.
100–299	0.153	1.25 (1.04–1.50)	0.218	1.08 (0.95–1.22)	0.006	1.00 (0.65–1.53)	0.006	1.07 (0.69–1.67)
300–499	0.321	2.63 (1.82–3.78)	0.254	1.25 (1.07–1.48)	≤0.018	—	—	—
≥500	0.563	4.60 (3.92–5.40)	0.298	1.47 (0.96–2.25)	≤0.017	—	—	—

^a^ Cells with counts ≤9 cannot be reported according to the cell size suppression policy of the database. The incidence proportion of those with cell counts ≤9 is displayed with the numerator equal to 9 along with the ≤ signs.

^b^ Adjusted for provider type, setting, prescriber, sex, age, individual antipsychotics, and chlorpromazine-equivalent dosage.

CI, confidence interval; IP, incidence proportion; IPR, incidence proportion ratio; Ref., reference.

## Discussion

To the best of our knowledge, this study is the first study outside the United States with the longest follow-up period, conducted to assess glucose and prolactin monitoring patterns among children and adolescents initiating antipsychotic therapy. We found that only 14% received a glucose test within 30 days before drug initiation. Our estimate for baseline glucose monitoring was similar to that (8%–16%) reported in previous studies that used threefold to sixfold wider time windows (Delate et al. 2014; Raebel et al. 2014; Connolly et al. 2015). We also found that 21% received a glucose test between 30 days before and 180 days after drug initiation. This estimate in our study was lower than that (32%) reported in a previous study of a financially poorer population (Morrato et al. 2010).

Our study extends these previous studies in several important respects. First, we demonstrated that the incidence proportion of those who received baseline monitoring for glucose was <30% in most subgroups, including olanzapine users who are considered at greater risk of type II diabetes (Galling et al. 2016). Second, we observed that only 25% received a glucose test through 450 days after drug initiation. Third, we found that only 0.9% consecutively received a glucose test at baseline, 1- to 3-month, 4- to 9-month, and 10- to 15-month periods. Fourth, we showed that the incidence proportion of those who received prolactin monitoring was much lower than those who received glucose monitoring.

The low incidence proportion of those who received glucose and prolactin monitoring in our study might be explained by physician, patient, and guardian characteristics. Previous studies have identified several barriers for metabolic monitoring in children and adolescents initiating antipsychotics (Walter et al. 2008; Ronsley et al. 2011; Rodday et al. 2015; McLaren et al. 2017). Low confidence in deciding the course of action in response to abnormal results may be one of the major barriers to conduct metabolic monitoring (Ronsley et al. 2011). Other common barriers are patient and guardian nonadherence and refusal to allow blood testing (Walter et al. 2008; McLaren et al. 2017). In addition, unavailability of guidelines for the use of antipsychotics specific to children and adolescents might be one of the barriers in Japan, although two associations in the United States and Canada have proposed such guidelines (American Academy of Child and Adolescent Psychiatry 2011; Pringsheim et al. 2011). To overcome barriers to conduct metabolic monitoring, there are several promising strategies, including formulation of a standardized metabolic monitoring protocol (Ronsley et al. 2011), education of physicians with regular audits and feedback regarding metabolic monitoring (Cotes et al. 2017), specialty certification that requires demonstration of practice performance improvement (Rodday et al. 2015), a pop-up alert system that prompts physicians to order laboratory testing (DelMonte et al. 2012), and an insurer-based prescription monitoring that requires peer review of the appropriateness of antipsychotic therapy (Maryland Medicaid 2011).

Our study has several limitations. First, our data did not allow us to determine whether the laboratory tests were requested by the physician prescribing antipsychotics for the purpose of monitoring or if they were for the purpose of an unrelated medical evaluation. This is because the database did not include reasons for ordering laboratory tests and for writing prescriptions. The time to initial monitoring demonstrated a gradual increase 180 days after drug initiation, which suggests that monitoring was ordered for the purpose of other medical conditions rather than antipsychotic therapy. Second, we could not determine patient adherence for fasting before the glucose test, although several guidelines recommend assessing fasting plasma glucose (American Academy of Child and Adolescent Psychiatry 2011; Pringsheim et al. 2011). Third, the disagreement on the definition of the baseline period between our study and a previous study limited the comparability of results. We defined the baseline as the period from 30 days before the index date until the index date, although a previous study defined the baseline as the period from 84 days before the index date until 14 days following the index date (Delate et al. 2014). We did not include time after the index date in the baseline period, because, based on our clinical experience, new users of antipsychotics who are not fasting are unlikely to receive a fasting plasma glucose test in the days immediately following drug initiation. Fourth, retrospective claims data did not capture the reason for nonadherence to glucose and prolactin monitoring. Fifth, our data did not include the claims solely covered by public funds, comprising ∼286,000 recipients (1.3%) of the population aged ≤19 years (Ministry of Health, Labour and Welfare 2015). Sixth, cohort effects may have resulted in an increase or decrease in the proportion of those who received monitoring. In the study period during the fiscal year 2014, there was only one approved antipsychotic (pimozide) for the treatment of mental disorders in children and adolescents; however, in 2016, risperidone and aripiprazole were approved for the additional indication of irritability associated with autism spectrum disorders in patients aged <18 years (Otsuka 2016; Janssen 2017). There is a possibility that prescriber characteristics have changed after the additional indication, leading to a change in monitoring practices.

## Conclusions

Our study shows that the monitoring for glucose and prolactin are infrequently performed in children and adolescents initiating antipsychotic therapy. Strategies for physicians, patients, and guardians are needed to overcome the barriers in conducting glucose and prolactin monitoring.

## Clinical Significance

Despite the evidence on the growing number of antipsychotic users and the potential increased risk of diabetes and hyperprolactinemia in the pediatric population, the incidence proportion of those who received monitoring for glucose and prolactin before and after drug initiation is remarkably low. Greater efforts are needed to improve the monitoring for metabolic abnormalities.

## Supplementary Material

Supplemental data

Supplemental data

Supplemental data

Supplemental data

Supplemental data
