# Supplementary material for: Glucose and Prolactin Monitoring in Children and Adolescents Initiating Antipsychotic Therapy
Source: J Child Adolesc Psychopharmacol. 2018 Sep 14;28(7):454–62. doi: 10.1089/cap.2018.0013 (PMC6154762; doi:10.1089/cap.2018.0013)
Supplement: Supplemental data [file Supp_Table1.pdf]

TABLE S1. Complete list of psychotropic medications

Drug	Equivalent dose for oral drugs (mg/day)	Equivalent dose for injection drugs (mg/day)	Equivalent dose for depot injection drugs (mg/day)
FGAs (21 drugs)			
bromperidol	2	—	—
chlorpromazine	100	33	—
clocapramine	40	—	—
fluphenazine	2	—	15
haloperidol	2	1	30
levomepromazine	100	25	—
mosapramine	33	—	—
nemonapride	4.5	—	—
oxypertine	80	—	—
perphenazine	10	2	—
pimozide	4	—	—
pipamperone	200	—	—
prochlorperazine	15	2.14	—
propericiazine	20	—	—
reserpine	0.15	—	—
spiperone	1	—	—
sulpiride	200	50	—
sultopride	200	—	—
tiapride	100	—	—
timiperone	1.3	0.19	—
zotepine	66	—	—
SGAs (7 drugs)		—	—
aripiprazole	4	—	100
blonanserin	4	—	—
olanzapine	2.5	2.25	—
paliperidone	1.5	—	18.75
perospirone	8	—	—
quetiapine	66	—	—
risperidone	1	—	10

FGA, first-generation antipsychotics; SGA, second-generation antipsychotics.
