# Supplementary figures and images for: Glucose and Prolactin Monitoring in Children and Adolescents Initiating Antipsychotic Therapy
Source: J Child Adolesc Psychopharmacol. 2018 Sep 14;28(7):454–62. doi: 10.1089/cap.2018.0013 (PMC6154762; doi:10.1089/cap.2018.0013)

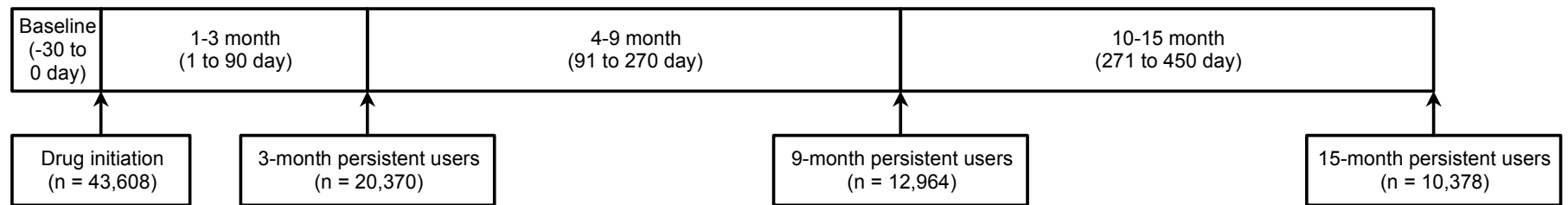

FIG. S1. Timeline for monitoring among persistent antipsychotic users.

Supplement: Supplemental data [file Supp_Fig1.pdf]

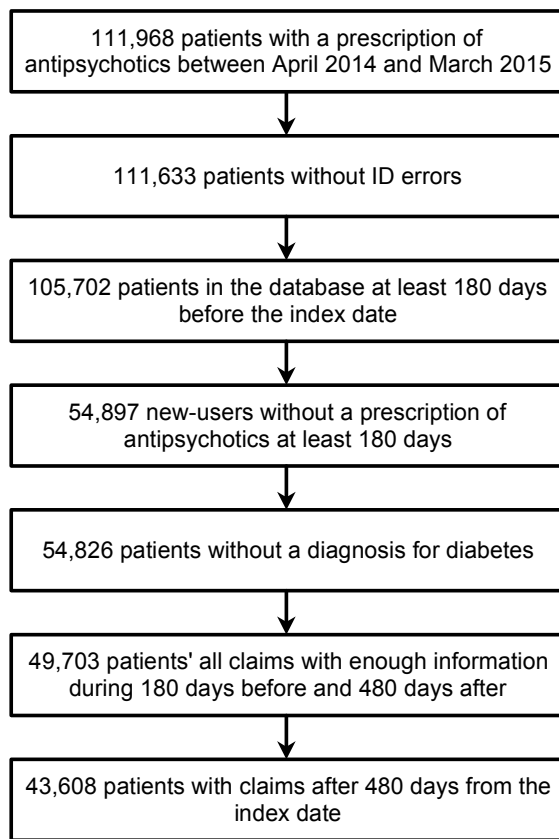

FIG. S2. Flow diagram.

Supplement: Supplemental data [file Supp_Fig2.pdf]

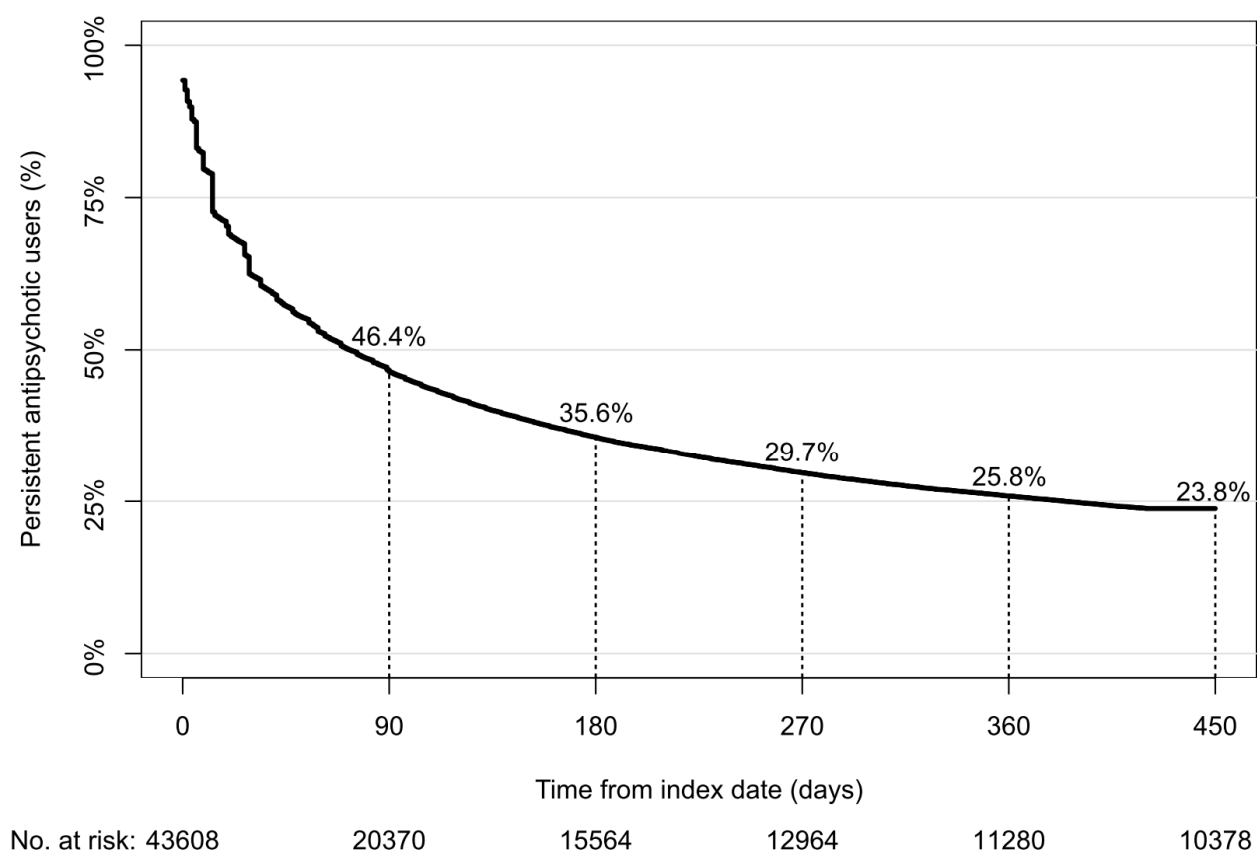

FIG. S3. Percentage of persistent antipsychotic users

Supplement: Supplemental data [file Supp_Fig3.pdf]
