# Supplementary material for: Glucose and Prolactin Monitoring in Children and Adolescents Initiating Antipsychotic Therapy
Source: J Child Adolesc Psychopharmacol. 2018 Sep 14;28(7):454–62. doi: 10.1089/cap.2018.0013 (PMC6154762; doi:10.1089/cap.2018.0013)
Supplement: Supplemental data [file Supp_Table2.pdf]

TABLE S2. Sensitivity analysis for incidence and correlates of metabolic and prolactin monitoring at baseline

	Glucose[a]				Prolactin[a]			
	Crude		Adjusted[b]		Crude		Adjusted[b]	
Characteristic	IP	IPR (95% CI)	IP	IPR (95% CI)	IP	IPR (95% CI)	IP	IPR (95% CI)
Provider type								
Clinic	0.102	ref	0.206	ref	0.004	ref	0.004	ref
Hospital	0.180	1.77 (1.39, 2.25)*	0.249	1.21 (0.95, 1.53)	0.007	1.66 (0.95, 2.90)	0.008	1.88 (0.95, 3.73)
Setting								
Inpatient	0.738	ref	0.502	ref	0.012	ref	0.008	ref
Ambulatory	0.097	0.13 (0.11, 0.15)*	0.102	0.20 (0.16, 0.26)*	0.005	0.43 (0.27, 0.67)*	0.004	0.45 (0.26, 0.78)*
Prescriber								
Non-psychiatrist	0.182	ref	0.231	ref	0.005	ref	0.005	ref
Psychiatrist	0.097	0.53 (0.44, 0.64)*	0.223	0.96 (0.79, 1.17)	0.006	1.39 (0.94, 2.05)	0.007	1.58 (1.02, 2.44)*
Sex								
Boys	0.112	ref	0.206	ref	0.003	ref	0.003	ref
Girls	0.166	1.49 (1.39, 1.59)*	0.249	1.21 (1.15, 1.26)*	0.009	3.19 (1.81, 5.60)*	0.010	2.80 (1.77, 4.42)*
Age, y								
0–3	0.494	2.90 (2.30, 3.65)*	0.230	0.82 (0.63, 1.06)	≤0.020	—	—	—
4–6	0.160	0.94 (0.58, 1.50)	0.229	0.81 (0.47, 1.39)	≤0.003	—	—	—
7–12	0.076	0.45 (0.34, 0.58)*	0.166	0.59 (0.44, 0.79)*	0.004	0.47 (0.22, 1.00)*	0.006	0.65 (0.32, 1.35)
13–15	0.134	0.78 (0.73, 0.84)*	0.243	0.86 (0.80, 0.93)*	0.005	0.64 (0.45, 0.90)*	0.007	0.69 (0.49, 0.98)*
16–18	0.171	ref	0.282	ref	0.008	ref	0.010	ref
Type of antipsychotics								
FGA	0.234	ref	0.322		0.006	ref	0.004	ref
SGA	0.076	0.32 (0.27, 0.38)*	0.165	0.51 (0.42, 0.63)*	0.005	0.77 (0.48, 1.25)	0.004	0.87 (0.53, 1.42)
Both	0.158	0.67 (0.50, 0.90)*	0.219	0.68 (0.53, 0.88)*	≤0.023	—	—	—
Chlorpromazine-equivalent dosage, mg								
0–99	0.122	ref	0.221	ref	0.006	ref	0.008	ref
100–299	0.153	1.25 (1.04, 1.50)*	0.216	0.98 (0.87, 1.10)	0.006	1.00 (0.65, 1.53)	0.006	0.79 (0.53, 1.18)
300–499	0.321	2.63 (1.82, 3.78)*	0.258	1.17 (1.01, 1.35)*	≤0.018	—	—	—
≥ 500	0.563	4.60 (3.92, 5.40)*	0.214	0.96 (0.82, 1.13)	≤0.017	—	—	—

* $p < .05$.

[a] Cells with counts ≤9 cannot be reported according to the cell size suppression policy of the database. The incidence proportions of those with cell counts ≤9 is displayed with the numerator equal to 9 along with the ≤signs.

[b] Adjusted for provider type, setting, prescriber, sex, age, type of antipsychotics, and chlorpromazine-equivalent dosage.

CI, confidence interval; FGA, first-generation antipsychotics; IP, incidence proportion; IPR, incidence proportion ratio; ref, reference; SGA, second-generation antipsychotics.
